# Cross-Split of Dislocations: An Athermal and Rapid Plasticity Mechanism

**DOI:** 10.1038/srep25966

**Published:** 2016-05-17

**Authors:** Roman Kositski, Oleg Kovalenko, Seok-Woo Lee, Julia R. Greer, Eugen Rabkin, Dan Mordehai

**Affiliations:** 1 Department of Mechanical Engineering, Technion – Israel Institute of Technology, 32000, Haifa, Israel; 2 Department of Materials Science and Engineering, Technion – Israel Institute of Technology, 32000, Haifa, Israel; 3 Department of Materials Science and Engineering & Institute of Materials Science, University of Connecticut, CT, United States; 4 Division of Engineering and Applied Science, California Institute of Technology Pasadena, CA 91125, United States

## Abstract

The pathways by which dislocations, line defects within the lattice structure, overcome microstructural obstacles represent a key aspect in understanding the main mechanisms that control mechanical properties of ductile crystalline materials. While edge dislocations were believed to change their glide plane only by a slow, non-conservative, thermally activated motion, we suggest the existence of a rapid conservative athermal mechanism, by which the arrested edge dislocations split into two other edge dislocations that glide on two different crystallographic planes. This discovered mechanism, for which we coined a term “*cross-split* of edge dislocations”, is a unique and collective phenomenon, which is triggered by an interaction with another same-sign pre-existing edge dislocation. This mechanism is demonstrated for faceted α-Fe nanoparticles under compression, in which we propose that cross-split of arrested edge dislocations is resulting in a strain burst. The cross-split mechanism provides an efficient pathway for edge dislocations to overcome planar obstacles.

The ongoing efforts to improve strength and ductility through miniaturizing specimens^1^ or by forming sub-micrometer scale microstructure^2^,^3^,^4^ bring with it the need to deeply understand mechanical properties of nanostructured materials on the atomic level, i.e. identify and relate the effect of dimensionality to properties of dislocations, line defects within the crystal structure^5^,^6^,^7^,^8^. In bulk material, dislocation motion is restricted by planar defects, such as grain boundaries and interfaces, which in turn lead to the material hardening^9^,^10^. This mechanism is exploited to increase the strength of material with sub-micrometer dimensions. For instance, the large portions of interfaces in bimetallic systems or the graphene-metal interface represent a planar barrier for dislocation motion^11^,^12^. However, dislocations may overcome these barriers by changing their slip plane, depending on their character. Screw dislocations can change their glide plane via a thermally-activated conservative process known as cross-slip. This process, which is temperature and strain-rate dependent, is prolific in BCC metals due to the fact that 〈111〉 screw dislocations can glide on any of the following slip systems: three {110} planes, three {112} planes and six {123} planes^13^. On the other hand, edge dislocations cannot cross-slip and are believed to overcome obstacles only by climbing, i.e., changing their glide plane by absorbing or emitting point-defect^14^. The rate of this non-conservative motion is dominated by vacancy diffusion, which makes it much slower mechanism than glide or cross-slip in BCC metals. At the nanoscale, the free surfaces are excellent sinks for vacancies, which makes dislocation climb much more onerous than cross-slip of screw dislocations.

Here we propose a rapid conservative athermal dislocation mechanism by which edge dislocations overcome obstacles and change their glide plane by splitting. This mechanism is a collective process by which edge dislocation splits into two edge dislocations which are not arrested by the obstacle. This mechanism is argued to be important in structures with low dimensionality and is demonstrated here to control the strength of α-Fe (BCC) nanoparticles under compression. We present here experimental results of BCC iron nanoparticles under compression. The nanoparticles, which are pristine of mobile dislocations prior to compression, undergo some strain hardening before deforming in a large strain burst. We show in molecular dynamics (MD) simulations that dislocations are nucleated at two of the upper vertices, from which two independent edge dislocation pile-ups are formed. While the nanoparticle hardens as the dislocation nucleation-driven pile-ups develop, the strain burst commences when the front dislocation in the pile-up splits into two edge dislocations on two different slip planes. This splitting allows the two newly formed dislocations to glide away from the interface, leading to the catastrophic deformation of the nanoparticle. We propose here that this splitting mechanism is not confined only to nanoparticles but is energetically favorable when the distance between the neighboring edge dislocations decreases below a certain threshold, i.e., this mechanism is unique since it is a process involving a group of dislocations.

Fe nanoparticles were formed via the solid-state dewetting method on a hard sapphire substrate. The formed particles are faceted with the top {110} facet being parallel to the substrate as shown in Fig. 1A. The nanoparticles were compressed from the top facet and the mechanical response was calculated (see Methods for details). The data in Fig. 1 conveys that at the early stages of the deformation, the nanoparticles were relatively compliant because the slope of the stress-strain data up to ~1.5% strain, which relates to the vertical displacement of 5 nm, corresponds to an average elastic modulus of 25 GPa. Such a low modulus correlates well with the mechanical properties of the hydroxide layer, as has been extracted from nanoindentation experiments (Sec. I in the Supplementary Information). The nanoparticles hardened up to compressive strains in the range of 4–5%, and the average slope in this regime increased to ~110 GPa. This value is substantially lower than what is expected for Fe, which we expect to be ~400 GPa for this geometry, as we shall further extract from MD simulations. The hydroxide layer is too thin to explain the lower slope at these strains, which correspond to the displacements of 13–17 nm. In addition, the hardening stage terminated in a ~1–3% strain burst; this behavior differs from the previously reported stress-strain response of pristine faceted nanoparticles, such as Au^15^ and Ni_3_Al^16^, where the nanoparticles deformed elastically and collapsed plastically after the first dislocation nucleation event with strain burst of almost 100%.

Strain bursts in bulk material usually correspond to dislocation avalanches, which give rise to an abrupt energy release within the dislocation microstructure^17^. In pristine nanoparticles the strain burst is attributed to the reduction in strength once the incipient nucleated dislocations escape the crystal at the free surface, leaving behind atomic steps^15^. The reduced slope during the initial stages of compression of Fe nanoparticles in this work and the non-catastrophic, limited initial strain burst suggest that the dislocation-driven deformation mechanism here is different than what has been reported for pristine nanoparticles^15^,^16^.

We performed MD simulations to gain insight into the physical origin of this markedly different mechanical response of Fe nanoparticles from all those reported before. The simulations revealed that the nanoparticle was initially compressed elastically with an effective modulus of E′ = 397 GPa. This modulus takes into account the geometry of the nanoparticle and the fact that we consider the stress at the top facet. A simple one dimensional model, which assumes a varying uniaxial strain along the nanoparticles height, shows that this value corresponds to an elastic modulus of E = 268 GPa, which is in good agreement with the values expected for Fe in the 〈110〉 direction. The model details are given in Sec. II in the Supplementary Information.

The simulations revealed that plasticity commences by nucleating dislocations at two opposite top vertices of the nanoparticle, which are at the cross section of the upper (110) facet and two {112} facets. At each vertex two possible slip systems, which can serve as dislocation nucleation sites, exist: 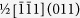 and 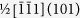 on the vertex marked (A) in Fig. 2a; 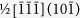 and 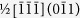 on the vertex marked (B). The directions in all cases are pointing inwards towards the substrate. Once nucleated, the dislocations are curved with two ends on the facets of the nanoparticle, obtaining mixed characters at both ends and an edge characteristic at its center. As the applied stress increased, the dislocations glided in the inward directions towards the substrate, and both ends traveled along the edges and the facets of the nanoparticles, straightening the overall dislocation line towards the edge character. In this mechanism, the dislocation line is eventually arrested close to the substrate, where it gained an edge character along its entire line. If the particle is sufficiently wide, the slip planes of the dislocations nucleated at vertices A and B do not intersect and they reach the substrate without interacting with one another. Since the dislocations are prevented from gliding by the interface, further deformation take place by additional similar nucleation events at the two vertices and inward propagation. The nucleated dislocations straightened themselves towards the edge character and glided towards the bottom of the nanoparticles, piling up behind the initial nucleated dislocations. This process repeated itself multiple times, with newly nucleated dislocations consecutively gliding away from the vertices towards the bottom of the nanoparticle, forming edge dislocations pile-ups along two independent 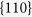 planes (Fig. 2b). These nucleation events did not produce catastrophic or substantial strain bursts, as was observed in FCC nanoparticles and Ni_3_Al^15^,^16^,^18^; after each nucleation event, the applied stress required to continue plastically deforming the nanoparticle increases (Fig. 3). Consecutive nucleation events require higher applied stresses in order to overcome the back-stress from the growing pile up, which manifests itself in hardening of the nanoparticle as it is compressed.

As long as only nucleation and glide is considered, the faceted shape of the nanoparticle and its crystallographic orientation coerced the emitted dislocations to pile against the substrate, without having the ability to interact in order to escape from their slip plane. As the deformation proceeded, it was necessary to nucleate additional dislocations at the vertices, which exacerbates the pile-up and requires the application of greater compressive forces. The stress cannot increase infinitely, so other, perhaps thermally-activated processes – like edge dislocation climb – may be activated to relieve the built-up stresses in the pile-up. Since all dislocations obtain an edge character, dislocation climb is the only known mechanism that can assist edge dislocation to escape out of the pile-up. However, this mechanism has a few shortcomings in rationalizing the experimental observations: it is a non-conservative process, which is expected to be sluggish at room temperature; the nearby free surfaces will drain vacancies and will make climb even slower; finally, even if climb takes place, it will not change the glide direction but rather translate slowly dislocations along the interface. Such a slow motion along the interface cannot rationalize the sudden displacement burst. Consequently, a different mechanism must be activated to enable the piled-up dislocations to escape while generating a massive and abrupt energy release.

We propose a novel path by which the pile-up can collapse and lead to a rapid energy release. In this mechanism, the edge dislocation at the head of the pile-up splits into two full edge dislocations on two different slip planes, which allow them to escape from the original slip plane, interact with the secondary pile-up, and lead to a catastrophic deformation of the nanoparticle. As more and more dislocations are nucleated into the pileup, the distance between the two leading dislocations within each pile-up decreased. At a certain compressive stress, which we shall further discuss, the dislocation at the head of the pile-up splits in to two dislocations, as demonstrated in Fig. 2c. In all our simulations the splitting started at the center of the nanoparticle, where the two segments of 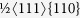 dislocations meet forming a short 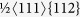 segment. The reaction in this direction allows for both the original and the split 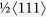 dislocations to lie on two different 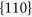 planes and share the same line direction. Starting at the center, the split dislocation continued along the two new 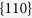 planes. Splitting was not limited to one of the pile-ups but we never observed it in both pile-ups simultaneously. Generally, the splitting rule can be written as follows


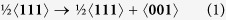


and is schematically shown in Fig. 4a,b. For instance, the pile-up marked in Fig. 2c is composed of 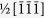 edge dislocation with two segments on the 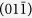 and 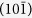 slip planes. The two dislocation parts form a short 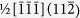 edge segment where the slip planes meet. This segment, at the head of the pile-up, splits into a 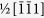 edge dislocation on the 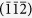 plane and a 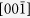 dislocation on the 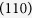 plane, which is parallel to the interface/upper facet. The 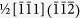 dislocation glided parallel to the second pile-up with the Burgers vector of the opposite sign, such that the Peach-Köhler force on the 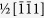 dislocation drives it away from the interface. In its motion upwards, the 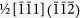 segment extends two “arms” into the two 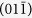 and 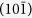 planes, splitting more edge segments in the original slip planes. As a result, the length of the 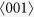 split segment increase and the 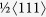 split dislocation glides upwards on the two 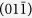 and 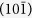 planes. This mechanism, which we denote as *cross-split*, allows arrested edge dislocations to “change” slip plane and to overcome barriers. This mechanism is a reminiscence of the well-known cross-slip mechanism of screw dislocations, in which the dislocation often change their glide plane. However, in contrast to cross-slip, the process reported here requires the edge dislocation to split and to leave a 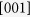 dislocation to fulfill the geometrical constraints of the Burgers vector. The newly formed 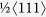 dislocation glided rapidly away from the pile-up, allowing a new dislocation to be nucleated into the pile-up without changing its density before cross-splitting. As the escaping dislocation reached the top interface, it forms an atomic step, which serves as a nucleation site for a 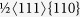 dislocation under compression. Finally, the newly nucleated dislocation glides and interacts with the second pile-up, which initiates a cascade of dislocation interactions that rapidly leads to the collapse of the pile-ups and to a decrease in the compressive stress over a wider range of strains. In conclusion, it is the cross-split of the dislocation at the head of one of the pile-ups that generates a rapid release of elastic energy stored within the nanoparticle during the compression, accompanied by the large strain burst seen in the experiments.

The outcome of the mechanism proposed here is somewhat similar to a group of reactions between dislocations and grain boundaries^19^,^20^,^21^,^22^. In the latter, the reaction between a dislocation and a grain boundary leads to formation of an interface dislocation (parallel to the interface) and the remaining dislocation is transmitted through or been reflected from the grain boundary. However, despite the similar outcome, there is a fundamental difference. In the simulations we consider the interface to be between two BCC lattices in the same orientation and the same lattice structure, but with different rigidities. Thus, any defect that is formed on such an interface will result in a mismatch between the atomic layers around the interface and will increase its energy, which makes the dislocation-interface reaction mechanism unfavorable. In addition, the dislocation-interface reaction mechanism does not require the proximity between two lattice dislocations, while we always observed the splitting when the core of two dislocations coalesced. A nice example for that is the formation of edge dislocation pile-ups in Si nanocubes under compression on a sapphire substrate^23^. The dislocations were found to be nucleated at the vertices and to pile-up against the interface. Nonetheless, at this stage the nanocube hardened since the dislocations did not interact with the interface, even at stresses in the GPa regime. Instead, parallel pile-ups were formed. Additionally, the difference between the compliances in the experiments between Fe (soft) and sapphire (hard) prevents dislocations to reach the substrate and penetrate into the sapphire, and these must remain as bulk dislocations inside the particle. Consequently, we propose that the mechanism shown here is a result of splitting due to coalescence of two dislocation cores. We defer further discussion on why this is an energetically favorable mechanism.

Results of our MD simulations suggest that in the strain range of 1.5%–5%, the experimental stress-strain data is unlikely to be purely elastic. At this stage of the deformation process, dislocation pile-ups are first formed. The emission of new dislocations into the pile-ups reduces the slope of the simulated stress-strain plot since each dislocation nucleation event contributes an additional incremental plastic strain, which is proportional to the Burgers vector. This type of deformation is regarded as pseudo-elastic rather than plastic^24^, despite the observed dislocation activity, because it is fully reversible, i.e. the release of applied compressive stress during the pile-up formation would result in dislocations gliding back to their nucleation sites. In that sense, it will be impossible to differentiate in the experiments between elastic and pseudo-elastic deformation, with a reduced slope. For instance, the stress vs. strain slope prior to the first nucleation event in the MD simulation of a 20.8 nm high nanoparticle is a factor of 2.4 higher than the average slope between the first nucleation event and the onset of cross-split (Fig. 3). If we take into account the additional strain accompanied with each dislocation nucleation, we find an effective pseudo-elastic modulus *E*_*eff*_ that satisfy


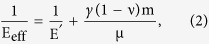


where *E′* is the effective modulus of the Fe nanoparticle extracted from the elastic part of the MD simulation in Fig. 3, μ and ν are the shear modulus and Poisson’s ratio, respectively, *m* is the Schmid factor and γ is a dimensionless factor that relates the number of dislocations in a pile-up to the particle’s height and the applied compressive stress. Based on the MD simulations we suggest that γ is equal to 0.89. Assuming isotropic elasticity theory, the slope of the simulated stress-strain plot decreases from E′ = 397 GPa to E_eff_ = 163 GPa, which is in a good agreement with the experimental slope. More details on the model are provided in Sec. II in the Supplementary Information.

Once the front dislocation in the pile-up cross-splits, it produces a strain burst, as was observed in the experiments at the end of the pseudo-elastic deformation. This strain burst was found to be relatively small, compared with those in other nanoparticles, which suggests that dislocations are not depleted from the nanoparticle but remain within the bulk and induce further hardening. A snapshot from the MD simulation after cross-split reveals a complex dislocation sub-structure (Fig. 2d). Once a dislocation cross-splits, it allows and facilitates the interactions between the two pile-ups in cascade-like fashion and leads to this complex microstructure. We believe that this is the reason for why the strain burst in the experiments is limited to a few percent of strain.

In contrast to cross-slip, in which no new dislocations are generated, the cross-split process adds a 〈100〉 dislocation to the system. It is natural to question whether the splitting would increase the energy of the system. The cross-split mechanism differs from previous mechanisms to overcome barriers because it is a collective mechanism, i.e., it is activated in the presence of other same-sign edge dislocations. Despite the increase in the number of dislocations, this mechanism reduces the total energy of the *whole* system subjected to the externally applied stress.

The cross-split is triggered by the overlap between the cores of two same-signed dislocations. The front dislocation is blocked on one side by the interface, and each subsequent dislocation is pushed towards the front one, as the stress is increased and more material is pushed downwards. As long as the edge dislocations cannot escape the pile-up, the distance between the two leading dislocations narrows, and at a certain stress level the cores of the two dislocations at the head of the pile-up will overlap (see Fig. 4c) and form an effective super-dislocation with a *2b*_111_ core, where *b*_*111*_ is the Burgers vector of the 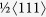 dislocation. This super-dislocation is unstable and prefers to dissociate into three dislocations, one on the original slip plane, with the original Burgers vector, and two others - on different slip planes, i.e., the reaction in the proposed cross-split mechanism. For example, the dislocations marked by “a” and “b” in Fig. 2c overlap under the applied stress and in result “b” remains on the original slip plane and a splits into “a_1_” and “a_2_”. The energy of the super-dislocation before cross-splitting is proportional to *(2b*_*111*_)^2^, which is higher than the energy after cross-split, proportional to 2*(b*_*111*_)^*2*^ + *(b*_*100*_)^*2*^ < *4b*_*111*_^*2*^, rendering the splitting is energetically favorable.

However, it is not sufficient for the core to split in order to reduce the super-dislocation energy, but the split dislocations have also to glide away from the original slip plane. To understand that, we calculate the Peach-Köhler force acting on the split 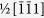 dislocation (marked as “a_2_” in Fig. 4b) after cross-splitting. The elastic forces are calculated under the assumption of isotropic elasticity theory^25^. For simplicity we consider only the two dislocations at the head of the pile-up that forms the super-dislocation. We assume that the 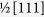 dislocation that remained on the pile-up plane (marked as b) is located where the splitting occurred. The 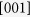 dislocation, marked as “a_1_”, is reported to be glissile in BCC metals^26^, albeit with a higher drag coefficient than 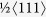 dislocations. Moreover, since their slip plane is parallel to the upper facet, the applied compressive stress does not contribute to the Peach-Köhler force acting on it. Thereupon, although it glides slowly towards the lateral surfaces due to the forces from the internal dislocation microstructure, we shall assume in the following analysis that the 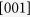 dislocation also remains at the original place of the splitting, while the split 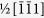 dislocation glides freely on its slip plane. As the split 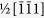 edge dislocation glides away from the pile-up, the force acting on it has three contributions: (1) dislocation “a_1_” attracts it back to recombine with itself, (2) dislocation “b” repels it, and (3) the applied compressive stress has a resolved shear stress component that pulls it away from the pile-up. The total force response, as a function of the distance travelled by the split 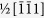 dislocation, is shown in Fig. 4d. At low stresses, the total Peach-Köhler force acting on dislocation “a_2_” is not sufficient to trigger splitting and to develop into the split-plane because of the substantial attractive force between the split dislocations. Even if splitting were to occur spontaneously, the elastic forces will keep all dislocations together. A higher compressive stress will increase the force pulling “a_2_” dislocation away from the pile-up, and when it reaches a resolved shear stress of about 6.5 GPa on the split plane, which corresponds to a compressive stress of ~13.7 GPa, the dislocations will spontaneously glide away from the pile-up.

Although these stresses are similar to the ones reached in the MD simulation when cross-split was identified (~11 GPa) one should not ascribe any significant importance to the exact value but rather as means to estimate to the orders of magnitude of stresses needed to pull the split products away from the pile. The simple force model does not take into account the contribution from all the dislocations in the pile-ups, nor does it account for the contribution of image forces from the surfaces and the interface; it was assumed the core interaction region is equal to *2b*_*111*_. However, under these high compressive stresses the core may be highly distorted and the core interaction region may increase substantially, which in turn will lower the threshold stress; Moreover, we considered classical elastic theory of dislocations with singularity at their core, which is sensitive at a distance of a few Burgers vector. All in all, we conclude that a stress of a few GPa is needed to drive the split dislocations away from the pile-up plane. These conditions are met in our case since these stress levels are required anyway to nucleate sufficient dislocation into the pile-up, so that the dislocation at the head of the pile-up will overlap. Thus, if cross-split occurs in the nanoparticles, the split dislocations will glide away from the pile-up plane.

It is worth noting that the MD simulations are performed in a low temperature and at a strain rate which is substantially higher than in the experiments. Zhu *et al.* showed that the stress needed to nucleate a dislocation on the surface is reduced by ~2.7 when considering room temperature and experimental strain-rate conditions^27^. Adapting the same ratio, we predict that an applied compressive stress of ~4.1 GPa is required in experimental conditions to nucleate the same number of dislocations in the pile-up as the one found in the MD at the onset of cross-split. These stress levels are in good comparison with the ones found experimentally at the strain burst (Fig. 1).

The cross-split mechanism is proposed here to responsible for the observed catastrophic deformation of α-Fe nanoparticles under compression, in which 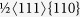 edge dislocations pile-ups are formed. However, these energy arguments given here for why splitting occurs are not limited to a particular slip system – if the stress conditions are sufficient for the core of two ½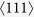 dislocations to overlap, cross-split may be triggered according to the dislocation reaction: 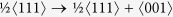. In Sec. III in the Supplementary Information we describe MD simulations of Fe nanowires under compression. In this geometry, 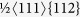 pure edge dislocations are nucleated and we show that cross-split occurs. The proposed cross-split mechanism provides an additional conservative mechanism for dislocations to escape from the interface and to allow further deformation, in addition to cross-slip and climb mechanisms, and it is believed to be important when the external dimensions of the samples are similar to the characteristic microstructural material length scale, at nanometer length scales. Understanding this mechanism enables tailoring overall mechanical properties via introduction of interfaces that will serve as obstacles for dislocation motion, such as in nanolaminates, multilayer nanocomposites, precipitate-hardened superalloys, graphene-metal nanocomposites etc.

## Materials and Method

### Nanoparticle Fabrication

Fe nanoparticles were formed via the solid-state dewetting method on a hard sapphire substrate. Fe film of 25 nm in thickness was deposited with the aid of magnetron sputtering on the 0.43 mm thick (0001)-oriented polished sapphire single crystal substrates that was ultrasonically cleaned with acetone, ethanol and de-ionized water before deposition. The deposition was performed with RF Magnetron sputtering tool, supplied by “von Ardenne”, using 99.9% pure Fe target by “Kurt J. Lesker” Company. The base pressure in the system and the Ar sputtering pressure were 3 × 10^−7^ Torr and 3 × 10^−7^ Torr, respectively. The thermal treatment at 850 °C for 24 h was performed in tube furnace in the flow of Ar + 10%H_2_ ultra-high purity gas, to prevent the oxidation and to cause a reduction of thin surface oxide film formed during samples manipulation in air. The samples were placed in the quartz boat and introduced into the hot zone of the furnace by a manipulator. The particles were characterized by Atomic Force Microscope (AFM) (XE – 70, Park Systems Corp.) operating in the tapping mode (NSG30 probes by NT-MDT). All nanoparticles had a faceted shape with a {110} upper facet parallel to the interface. TEM analysis revealed a thin, ~5 nm-thick hydroxide layer on the surface of the nanoparticles (Fig. S1 in the Supplemental Information).

### Compression of Nanoparticles

**Figure 1:**
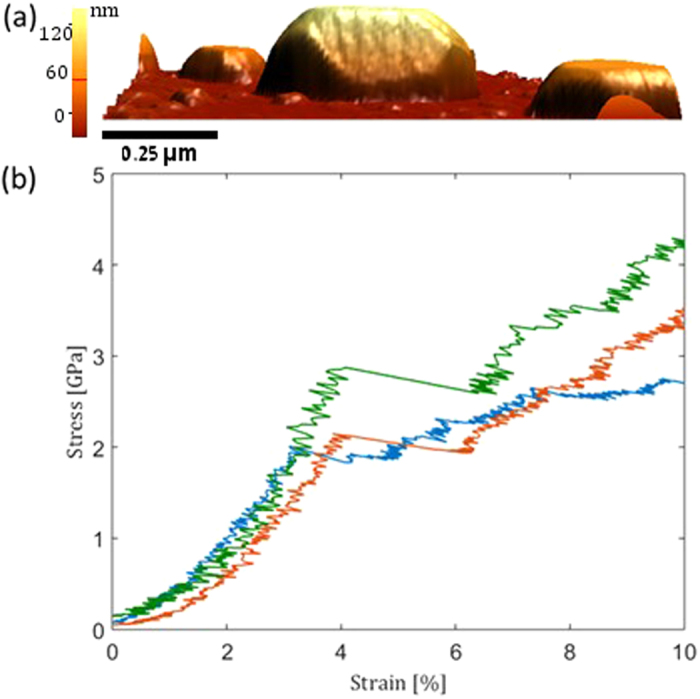
Fe nanoparticles under compression. (**a**) 3D AFM image of as-fabricated Fe nanoparticles produced by the dewetting technique. All Fe nanoparticles are oriented with a 〈110〉 axis perpendicular to the substrate. (**b**) Stress-strain data of three different Fe nanoparticles compressed along the 〈110〉 direction. The height of each nanoparticle is ~340 nm.

The nanoparticles were compressed and their mechanical response was calculated. We used *in-situ* nanomechanical testing system (InSEM^TM^, Nanomechanics, inc.), equipped in scanning electron microscope (Quanta, FEI) to measure load-displacement data. Uniaxial compression tests were performed with the flat punch diamond tip with the diameter of 1.5 μm. The constant nominal displacement rate of 5 nm sec^−1^ was used. Fig. 1b shows several representative stress-strain curves. Due to the faceted shape of the nanoparticles, the stress was defined as the engineering compressive stress acting on the top facet and the engineering strain was the relative change of height.

### Molecular Dynamics Simulations

The Molecular Dynamics (MD) simulations were carried out using the Large-scale Atomic/Molecular Massively Parallel Simulator (LAMMPS)^28^. The interatomic interactions are described according to the embedded atom method (EAM). We used a potential developed by Mendelev^29^ which was found to well reproduce surface energies as well as the plastic deformation of Fe nanoparticles^30^. We have also performed trial simulations using the Ackland^31^ EAM potential and have observed the cross-split process (see Fig. S7 in the Supplementary Materials). We also employed the and MCM2011^32^ EAM potential, but during compression dislocations were nucleated also on {001} planes, which we could not justify^30^. The faceted shape was carved from a BCC perfect lattice according to the Winterbottom construction^33^, based upon the relaxed surface energies calculated via MD at 10 K. The sapphire substrate was modeled using frozen atoms in the same lattice configuration as the particle. The interface energy between the substrate and the particle was estimated using the Winterbottom construction in order to reproduce the particle shape found experimentally^34^. The nanoparticles were first relaxed using the conjugate gradient (CG) method, followed by 80,000 molecular dynamic time steps of 5 fs each. Compression was accomplished using a virtual plane moving along the z [110] direction at velocity of 1 m/s. During the indentation, the temperature was kept on average to be 10 K using the NVT thermostat. Atom visualization and detection was carried out using the Atomeye^35^ and Ovito software (includes the dislocation extraction algorithm)^36^.

## Additional Information

**How to cite this article**: Kositski, R. *et al.* Cross-Split of Dislocations: An Athermal and Rapid Plasticity Mechanism. *Sci. Rep.* **6**, 25966; doi: 10.1038/srep25966 (2016).

## Supplementary Material

Supplementary Information

## Figures and Tables

**Figure 2:**
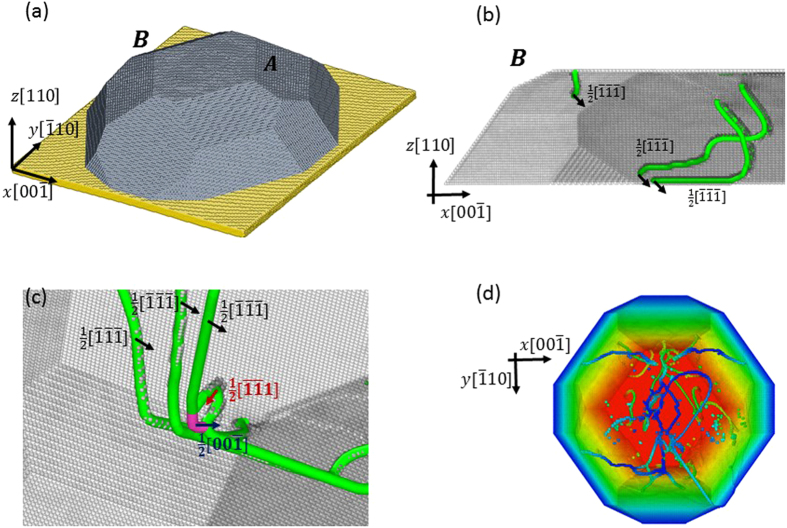
A 20.8nm high Fe nanoparticles under compression in the MD simulations. (**a**) An isometric view of the nanoparticle as modeled in the MD. The two top vertices marked with “A” and “B” are the points where dislocations nucleate. (**b**) A view from the 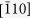 direction on cross-section of the nanoparticle. A three-dislocation pile-up formed at B is shown, where all the atoms were removed except those in the dislocation cores. (**c**) A view from the bottom on the dislocation pile-up shown in (b) slightly after the front ½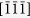 dislocation in the pile-up splits into 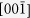 and ½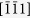 dislocations. For clarity, an elongated nanoparticle in the 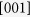 direction is shown. (**d**) is a bottom view into the particle after cross-split. The color represent height with red being the height of the top facet and blue - the bottom. Some vacancies are left as remnants to dislocation-dislocation interactions.

**Figure 3:**
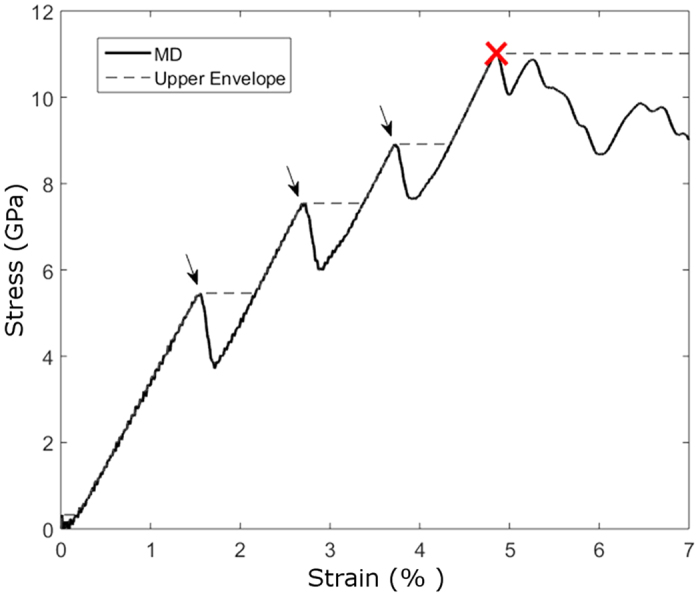
Simulated stress vs. strain of a 20.8 nm high nanoparticle under compression. The first three yield drops, marked by arrows, correspond to nucleation of dislocation and to the formation of the pile-up. The yield drop at the onset of cross-split is marked with a cross. In the force-control interpretation (the upper envelope curve) suggested in^15^, the strain burst is associated with the cross-split at the head of the pile-up.

**Figure 4:**
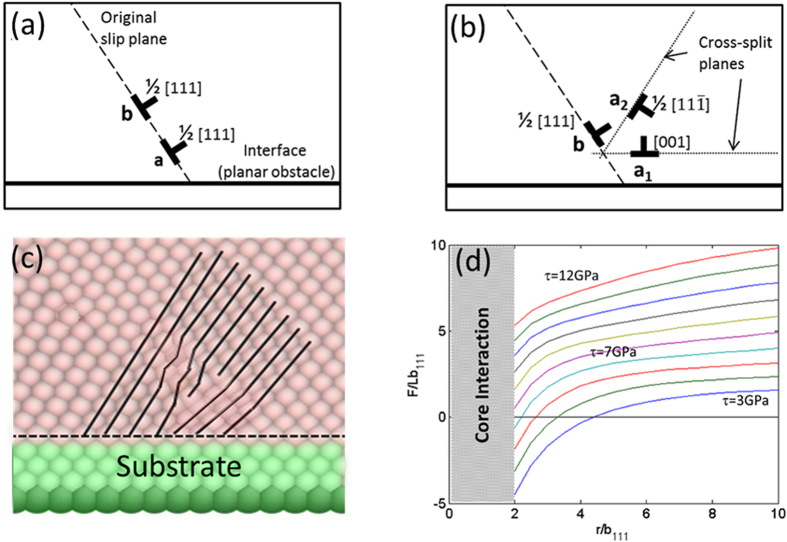
The cross-split mechanism. (**a**) Two dislocations at the head of a pile-up prior to the cross-split. (**b**) Following the splitting of the leading dislocation marked with the letter “a”, two newly formed dislocations are released from the pile-up: “a_1_” and “a_2_”. (**c**) The overlap between the cores of the two front dislocations before cross-splitting in the MD simulation. To facilitate the identification of the dislocation cores, planes of atoms in perpendicular to the slip plane are marked with full lines. (**d**) The component of the force acting on the split dislocation marked as “a_2_” along its slip direction as a function of the distance from the pile-up *r*, for various resolved shear stresses on the split plane. Negative and positive force points towards and away from the pile-up, respectively.
