# Supplementary material for: Cross-Split of Dislocations: An Athermal and Rapid Plasticity Mechanism
Source: Sci Rep. 2016 May 17;6:25966. doi: 10.1038/srep25966 (PMC4869067; doi:10.1038/srep25966)
Supplement: Supplementary Information [file srep25966-s1.pdf]

Supplementary Materials:

Cross-Split of Dislocations: An Athermal and Rapid Plasticity Mechanism

Roman Kositski[1], Oleg Kovalenko[2], Seok-Woo Lee[3], Julia R. Greer[4], Eugen Rabkin[2] and Dan Mordehai[1]

I. Strength of the Hydroxide Layer

The nanoparticles are formed by annealing in the tube furnace under the constant flow of Ar +10 vol.% H_2 ultra-high purity (UHP) gas. This gas atmosphere is chosen to prevent the oxidation and to reduce the thin oxide film on the surface formed during samples manipulation in air. Transmission Electron Microscopy (TEM) images of a cross-section of a nanoparticle (Fig. S1) shows that such a thin-layer of hydroxide is formed and its thickness varies in a range of a few nanometers. We estimate its thickness to be around 5 nm on average.

To check the strength of the hydroxide layer that forms during exposure of Fe particles to the air, several samples were annealed at 400 ˚C for 4 h in air in order to form fully oxidized nanoparticles (see Fig. S2A). For comparison, we show in Fig. S2B the faceted nanoparticles, which are compressed in this work. Both types of nanoparticles are indented in Atomic Force Microscope (AFM) with the aid of NanoIndentation 2.0 module for XEP 1.7.70 data acquisition software (Park Systems Corp.). The probe used in experiment is nanoindenter diamond tip attached to a sapphire cantilever provided by Micro Star Technologies. The indentation was performed in load control mode employing 1 μN/s loading/unloading rate.

Representative force-displacement curves of the indentation of faceted and oxidized nanoparticles are given in Fig. S2C. The force on Fe nanoparticles is increasing with the displacement and a strain burst occurs at an indentation depth of about 10 nm. The resulting curve of the oxidized nanoparticle is monotonously increasing and do not exhibit displacements burst as in the case of Fe particles. Moreover, for the same applied load, the indenter penetrates much deeper into the oxidized nanoparticle than into the metallic Fe particle, indicating that Fe oxide is much softer than Fe. This fact implies that the hydroxide on the surface of Fe nanoparticles deforms much easier than the underlying crystal. Indeed, this difference in strength is reflected in the curve of the faceted Fe nanoparticle in Fig. S2C. The curves of the non-oxidized and oxidized Fe nanoparticles converge only at low penetration depths below 5 nm (the

thickness of the oxide layer), which means that the mechanical response of the Fe particles is dominated by the surface hydroxide layer at the lower depths.

Detailed examination of the AFM images acquired after the indentation of Fe particles demonstrated that some particles did not yield plastically under applied load. The shallow residual imprints on the upper facet of such particles have a form of the triangle but the depth of the imprints is only about 6-7 nm instead of tens of nanometers, while the maximum penetration depth of the tip was a few tens of nanometers. We relate these shallow indents with Fe oxide that was easily pushed to the sides of the imprint during indentation. In contrast, the underlying Fe crystal completely recovered its initial form after unloading.

Based upon these observations we conclude that the oxide layer is much weaker than Fe and that the contribution of the oxide layer to the force measured during indentation of Fe non-oxidized nanoparticles is negligible. Thus, we can approximate the indentation force of the Fe nanoparticles to be elastic and to fit a Hertzian contact model to the indentation force *F* in Fig. S2C

$$F(d) = E^{*}\sqrt{Rd^{3}} \qquad \text{(S1)}$$

where E^* is the reduced elastic modulus, *d* is the indentation depth and *R* is radius of the tip. For comparison, we fit the similar model to the oxidized nanoparticles. We note that the strength of the oxidized nanoparticle is low and the deformation is not elastic but plastic. The large plastic deformation of the thin oxide layer of the nanoparticles leads to the conclusion that the elastic part is negligible in the curves of the oxidized nanoparticle. Therefore, the reduced modulus is not an elastic modulus but an estimated effective plastic modulus that relates plastic stress and strain. This modulus is the one important for our discussion, since this is the relevant modulus when compressing the Fe nanoparticles with a flat punch.

The Hertzian fit to the results yields the following values for the Fe and oxidized nanoparticles:

$$E^{*}_{Fe}\sqrt{R} = 926 \pm 118\; GPa\, nm^{0.5} \qquad \text{(S2)}$$

$$E^{*}_{oxide}\sqrt{R} = 129 \pm 3\; GPa\, nm^{0.5} \qquad \text{(S3)}$$

The reduced moduli are defined as

$$\frac{1}{E^{*}_{x}} = \frac{1-\nu_{tip}^{2}}{E_{tip}} + \frac{1-\nu_{x}^{2}}{E_{x}} \qquad \text{(S4)}$$

where *x* stands for *Fe* or *oxide*. The diamond tip has an elastic modulus of $E_{tip}=1141\ GPa$ and Poisson's ratio of $\nu_{tip}=0.07$[1]. We also consider that the elastic properties of Fe fulfill $E_{Fe}=211$

GPa and $\nu = 0.29$ [2]. Since the strength of the hydroxide layer is low, we postulate ν_{oxide} to be equal to 0.5, which is a typical value for a non-compressible solids. Employing these elastic properties in equations (S2)-(S4) we find that R=23.3 nm and E_{oxide}=20.5 GPa.

The radius of the tip obtained in this analysis is in good agreement with experimental observations. Detailed AFM scans of the residual indents in Al single crystal was performed and the radius of curvature at the residual indent was in the range of 20-30 nm. More importantly, the effective elastic modulus of the oxide is an order of magnitude smaller than that of Fe.

II. The Pseudo-Elastic Deformation

At the early stages of the deformation, the deformation is expected to be concentrated in the hydroxide layer since it is much softer than the Fe part. Since the hydroxide layer is about 5 nm thick, his part of the deformation corresponds to a compressive strains of up to 1.5% in Fig. S3. Indeed, the slope at this stage agrees with elastic modulus of 25 GPa, which is an order of magnitude lower than the elastic modulus of Fe. This value is in a very good agreement with the estimated values from the nanoindentation given in Sec. II in this Supplemental File.

For compressive strains larger than 1%, it is fairly reasonable to assume that the mechanical response is mainly from the Fe part, due to the large differences in the compliances of the hydroxide layer and the Fe nanoparticle. Nonetheless, even for strains above 1%, the slopes in the stress-strain curve are substantially smaller than the expected elastic response. To demonstrate the theoretical curve, we plot in Fig. S3 the linear response with an effective elastic modulus equal to E'=397 GPa, as has been extracted from the Molecular Dynamics (MD) simulation shown in Fig. S4. We note that this elastic constant is an effective value, as it relates to the compressive stress on the top facet of the faceted nanoparticle and the average compressive strain.

The effective elastic modulus can be estimated with a simple one dimensional model, which assumes a varying uniaxial strain along the nanoparticles height. Let us assume that the nanoparticle is constructed of thin cylinders piled on top of another, with a varying cross-section area $A(x)$, parallel to the substrate and top surface. x is the distance from the top of the nanoparticle. Assuming that force F acts on the top facet of the area $A(0)$, the total elastic compression of the nanoparticle Δx is

$$\Delta x = \int_0^h \frac{F}{A(x)E} dx, \qquad \text{(S5)}$$

where h is the particle height and E is the elastic modulus of Fe in the <110> direction. The faceted shape of the nanoparticle is complex and for simplicity we assume the diameter is increasing linearly along the height,

$$A(x) = A(0) + \left(\frac{x}{h}\right)^2 \left(A(h) - A(0)\right). \qquad \text{(S6)}$$

A(0) is the cross section of the top facet and *A(h)* is the cross section area of the bottom surface just above the substrate. In the faceted nanoparticle, the largest cross-section area is three times larger than the area of the top facet. Therefore, we shall consider that

$$A(h) = 3A(0). \qquad \text{(S7)}$$

Combining equations (S5)-(S7) we find that

$$\Delta x \approx \frac{F}{E}\frac{0.675h}{A(0)} \qquad \text{(S7)}$$

The effective modulus *E'* is defined as the applied compressive stress *F/A(0)* divided by the engineering strain $\Delta x/h$ and it satisfys the relation

$$E' = \frac{F}{A(0)}\left(\frac{\Delta x}{h}\right)^{-1} \qquad \text{(S8)}$$

From equations (S7) and (S8) we find that

$$E' \approx 1.48E \qquad \text{(S9)}$$

Equation (S9) suggests that the effective elastic modulus is ~50% higher than *E*. Despite the simplicity of this approach, for typical values for the elastic modulus in the <110> directions, which are in the range 240-260 GPa (24), the effective elastic modulus from equation (S9) is in the range *E'*=355-385 GPa, which is in good agreement with the MD results.

We suggest here that dislocations are nucleated at this pseudo-elastic deformation stage, which leads eventually to the large strain burst. We propose that the nanoparticles deforms in a pseudo-elastic fashion, i.e., dislocation are nucleated during the deformation but when the load is removed, they are drawn back to their nucleation sites, leaving the specimen with no residual plastic strain. Such a behavior was predicted in the MD simulations in the main text, where dislocations nucleate and form a pile-up. The pile-up is stable only under the applied compressive stress. Once the stress is removed, the dislocations glide back to the vertices, leaving the nanoparticle defect free and with no residual deformation, like it was observed in some of our nanoindentation tests.

In this pseudo-elastic deformation, isolated dislocation nucleation events will not be visible in the experimental stress-strain curves, since the nucleation itself does not release a substantial amount of elastic energy. This is clearly demonstrated in MD simulation, where the load-control interpretation of the results leads to small load bursts (the upper envelope in Fig. S4), followed by an increase in the compressive stresses. However, each nucleation will contribute to total strain and will decrease the effective slope of the stress-strain curve. For instance, the average slope of the stress-strain curves in the MD simulations presented in Fig. S4, once dislocations are being nucleated into the pile-up, decreases by a factor of 2.4 to 164 GPa.

The MD simulations suggest that two independent pile-ups are formed from the vertices along (110) slip planes. If N dislocations are nucleated into each pile-up, then the total pseudo-elastic strain (the strain that corresponds to dislocations nucleation only) $\varepsilon_{p\text{-}e}$ is

$$\varepsilon_{p-e} = \frac{N\, b \sin\theta}{h}, \tag{S10}$$

where h is the height of the nanoparticle and θ is the angle between the normal to the upper facet and the slip plane (in this work, θ=54.7 deg). The number of dislocations nucleated into the pile-up can be estimated from a continuous model of a stressed single pile-up[3]. The dislocation density in a stressed pile-up, bounded within the region $-l<x<l$ is

$$n(x) = \frac{2(1-\nu)\tau}{\mu b}\sqrt{\frac{l-x}{l+x}} \tag{S11}$$

where τ is the resolved shear stress on the slip plane in the glide direction, μ is the shear modulus and ν is Poisson's ratio. In the case of a nanoparticle, $\tau=m\sigma$ where $m=\sin\theta\cos\theta$ and σ is the compressive stress, and the length of the pile-up is equal to $l=h/\sin\theta$. Thus, the total number of dislocations nucleated into the pile-up is

$$N = \int_0^{l} n(x)\,\mathrm{d}x = \gamma\frac{(1-\nu)hm\sigma}{\mu b\sin\theta} \tag{S12}$$

where $\gamma=2+\pi$. From equation (S10) and (S12) we find that

$$\varepsilon_{p-e} = \gamma\frac{(1-\nu)\mathrm{m}\sigma}{\mu} \tag{S13}$$

The total compressive strain ε is the sum of the elastic and pseudo-elastic strains, $\varepsilon_e=\sigma/E$ and $\varepsilon_{p\text{-}e}$, respectively. Therefore, the slope of the stress-strain do not correspond to the elastic modulus E but to an effective value $E_{eff}=\sigma/\varepsilon$ that satisfies

$$\frac{1}{E_{eff}} = \frac{1}{E} + \frac{\gamma(1-\nu)m}{\mu} \tag{S14}$$

Let us consider the following values: E=397 GPa, μ=82 GPa, ν=0.291, and m=0.47. We recall that E depends on the shape of the nanoparticle, while the values of shear modulus and Poison's ratio are material properties. Therefore we consider the values as independent and we do not relate between them. The effective elastic modulus is then E_{eff}=42 GPa. This value is indeed consistent with a reduction in the measured slope in the stress-strain curves, as was found experimentally.

However, despite the reduced predicted slope, the calculated effective modulus is not in accordance with the MD simulations in Fig. S4. We attribute it to the accuracy of equation (S11) in small dimensions. In nanoparticle heights of a few tens of nanometers, only a few dislocations are nucleated into the pile-up and the continuous dislocation density function given in equation (S11) overestimates number of dislocations. For instance, equation (S12) suggests that in order to add a dislocation into the pile-up, the compressive stress should increase by

$$\Delta\tau = \frac{\mu b \sin\theta}{\gamma(1-\nu)mh} \tag{S15}$$

If we consider the nanoparticle in the MD simulations, which is 20.8 nm high, we find that $\Delta\tau$ = 0.46 GPa, while in the MD an increase of 1.9 GPa in compressive stress was needed to nucleate new dislocations into the pile-up.

To better estimate the pseudo-elastic deformation for small number of dislocation, we suggest that the expression for N given by equation (3S) describes correctly its dependence on material properties and external stress, but with different value for γ. For the compressive stress to increase at a rate of 1.9 GPa per dislocation nucleation in a 20.8 nm high nanoparticle, γ should be equal to 0.89 (as opposed to 2+π). With this value of γ we find that the effective elastic modulus is E_{eff}=163 GPa, which is in excellent agreement with the effective slope during the pseudo-elastic deformation in the MD simulations.

Moreover, for a 350 nm high nanoparticles, the model predict that the stress increments between dislocation nucleation into the pile-up is $\Delta\tau$=0.16 GPa. In the range between a strain of 1% and the strain burst, where we suggest that hydroxide layer contribution is negligible, the compressive stress increases by 2.5 GPa, i.e., between 15 and 16 dislocations are nucleated into the pile-up. This number of dislocations corresponds to a pseudo-elastic strain of about 1%. This

value is in agreement with the difference between the experimental curve and the elastic estimation (without the pseudo-elastic part) at the stress at which the strain burst occurs.

III. Cross-split in other slip systems

The elasticity model for cross-split is indifferent to the slip plane. In Fe, the predominant slip systems at room temperature are ½<111>{110} and ½<111>{112}. The dislocations in the former slip system were nucleated in our MD simulations for nanoparticles. However, dislocations were shown to undergo cross-split along segments which are connecting dislocations on two different {110} planes. We identified that the connecting segments lie on {112} planes. Thus, we wish to check whether the cross-split mechanism occurs if the edge dislocations pile up only on {112} planes. For this purpose, we constructed a faceted nanowire instead of a nanoparticle, with an axis along the $\langle\bar{1}10\rangle$ direction. A representative atomic configuration is shown in Fig. S5. The nanowire along the *y* axis was modeled in a similar fashion to the faceted nanoparticle: a thin slice, one lattice spacing thick, was generated employing a two-dimensional Winterbottom construction; in order to produce a wide enough nanowire the wire was stretched along the *x* direction inserting 20 lattice unit cells. Along the *y* axis a periodic boundary conditions were imposed, resulting in an infinite long nanowire. The periodic boundary conditions along the nanowire axis force ½<111>{112} dislocations to be nucleated at the vertices.

The simulations shows that edge dislocations are nucleated at the two upper edges of the nanowire and form two non-intersecting pile-ups on {112} planes. In Fig. S6 we show the dislocation pile-up at the late stages of its formation, where it is constructed of three edge dislocations in a nearly planar configuration. Since the dislocation sense vector is arbitrary ξ, we chose its direction so that $\xi \times b$ is a vector pointing towards the extra semi-infinite plane of atoms from one side of the slip plane. Given that the arbitrary sense was chosen, the pile-up marked in Fig. 6 consists of ½$[\bar{1}\bar{1}1]$ edge dislocations. As we further compress the nanowire, the ½$[\bar{1}\bar{1}1]$ dislocation at the head of the pile-up cross-splits into two dislocations. One dislocation is a ½$[\bar{1}\bar{1}\bar{1}]$, which has the same edge characteristic as the original ½$[\bar{1}\bar{1}1]$ one but on a rotated {112} plane. The second formed dislocation is a $[001](110)$ dislocation, as in the nanoparticle. After cross-splitting, the ½$[\bar{1}\bar{1}\bar{1}]$ edge dislocation quickly glides away from the interface. The $[001]$ dislocation is also reported to be glissile in BCC metals[4], although with a higher drag

coefficient. Indeed, it glides slowly towards the lateral surfaces, eventually leaving the nanowire. This allows room for the next dislocation in the pile-up to glide forward and to become the front dislocation.

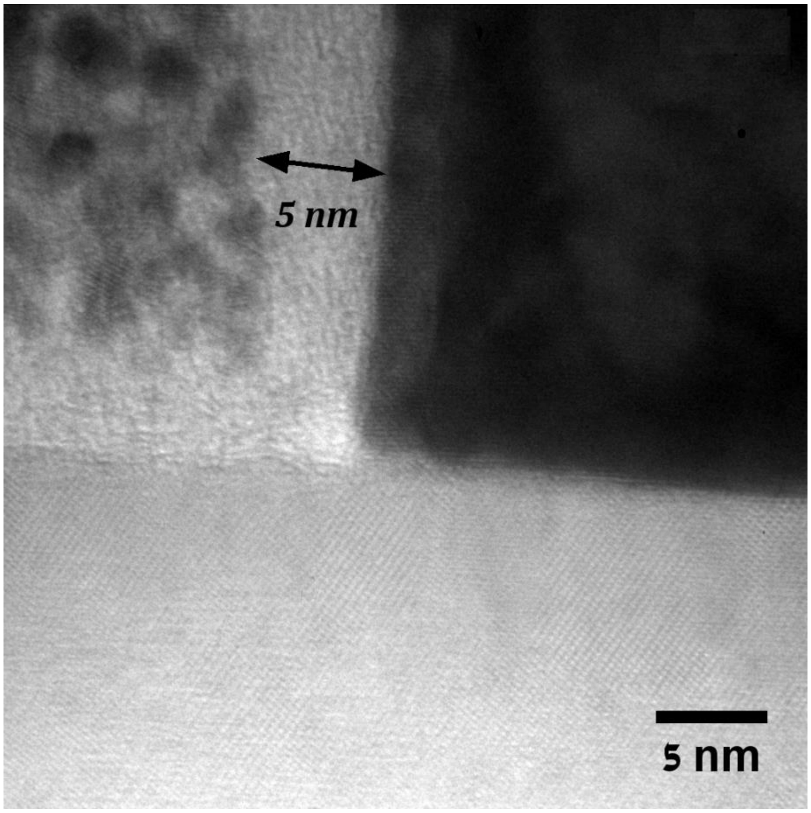

Fig. S1. A TEM cross-section of a nanoparticle. The sapphire substrate is in the lower part of the image. The nanoparticle is on the left side of the image and a thin hydroxide layer of about 5 nm is marked in the image.

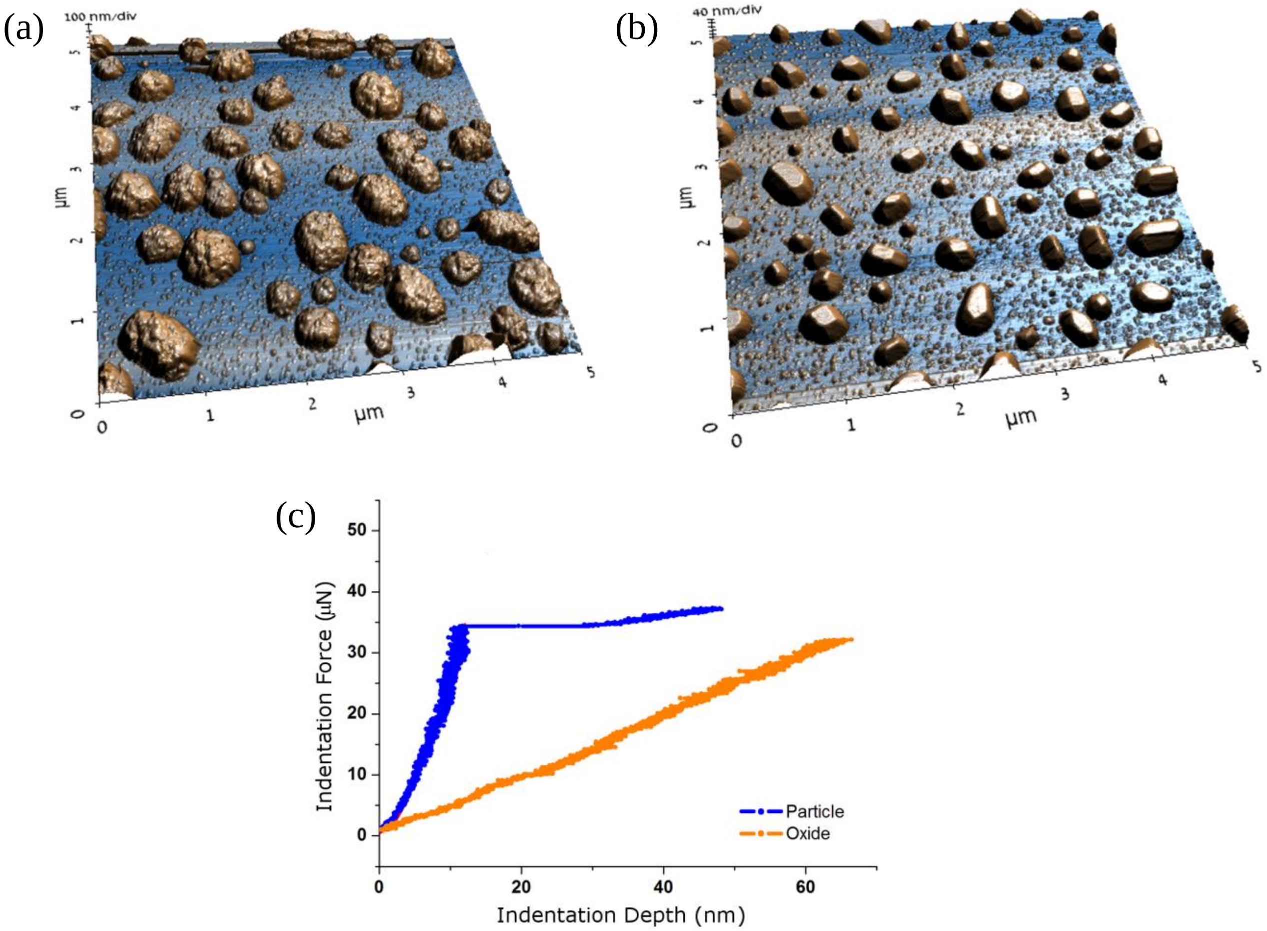

Fig. S2. Nanoindentation of Fe nanoparticles. (**a**) An AFM image of oxidized Fe nanoparticles. (**b**) The faceted Fe nanoparticles after dewetting. (**c**) Typical force-displacement curves of faceted and oxidized Fe nanoparticles.

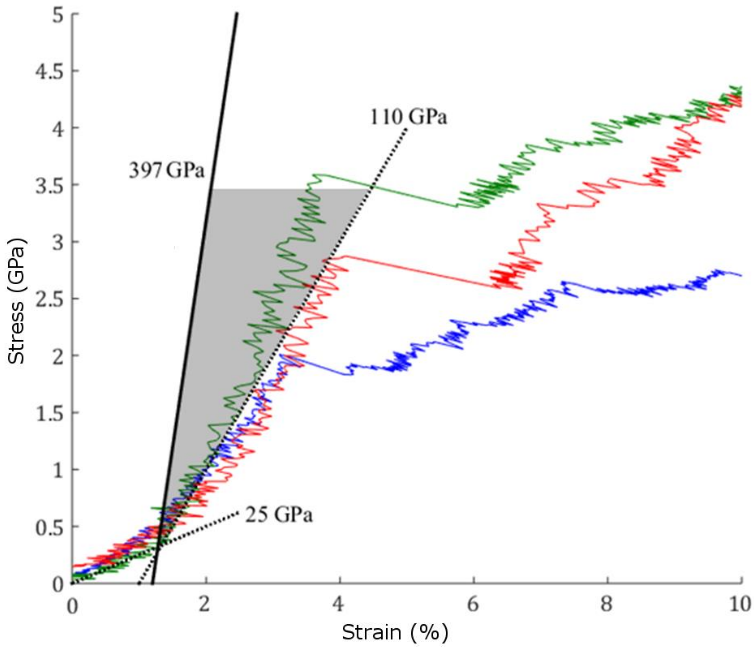

Fig. S3. The experimental stress-strain curves. The average slopes at strains below 1% and above 1% are marked in dashed lines. The corresponding effective elastic moduli are marked near the linear curves. For comparison, the expected elastic slope of pure Fe nanoparticle, with an effective elastic modulus extracted from the MD simulation, is plotted as a solid line.

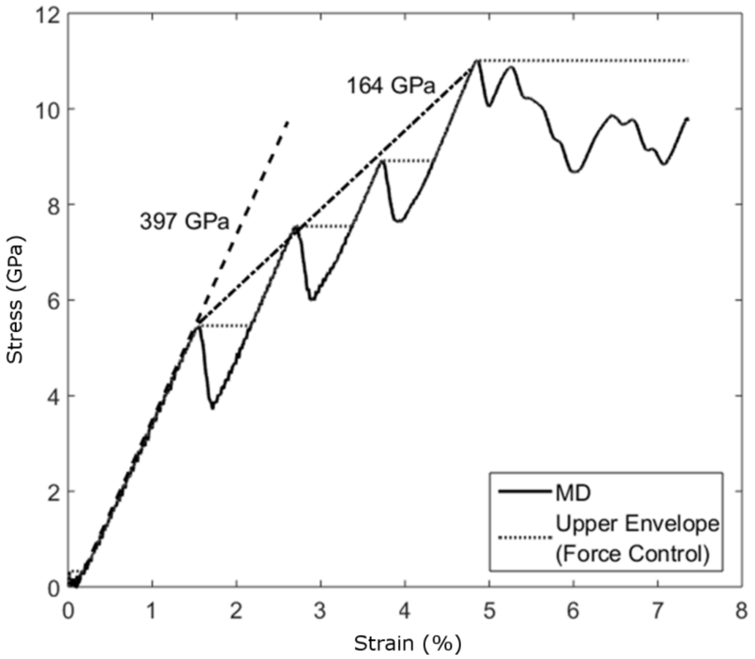

Fig. S4. Stress-strain curve of a 20.8nm high nanoparticle under compression. The same curve appears in Fig. 3 in the manuscript. The fully elastic stage is marked by a dashed line and its slope is marked on the plot. The pseudo-elastic effective modulus is also marked near the effective linear slope (dashed-dotted line).

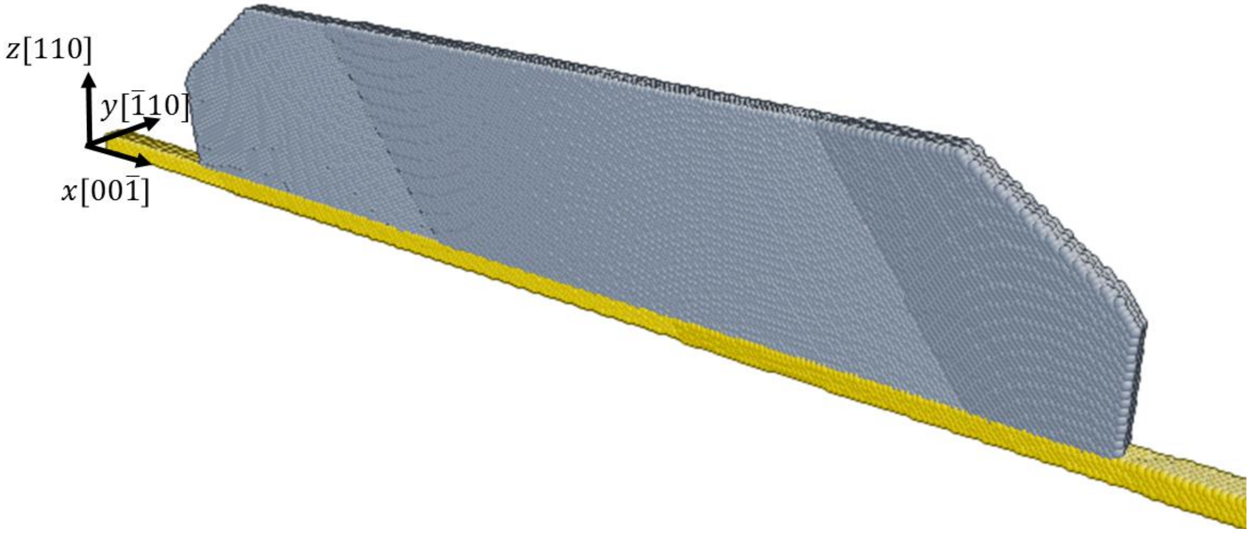

Fig. S5. Isometric view of the nanowire as modeled in the MD. The cell dimension along the nanowire includes only one unit cell whereas periodic boundary conditions are imposed in this direction.

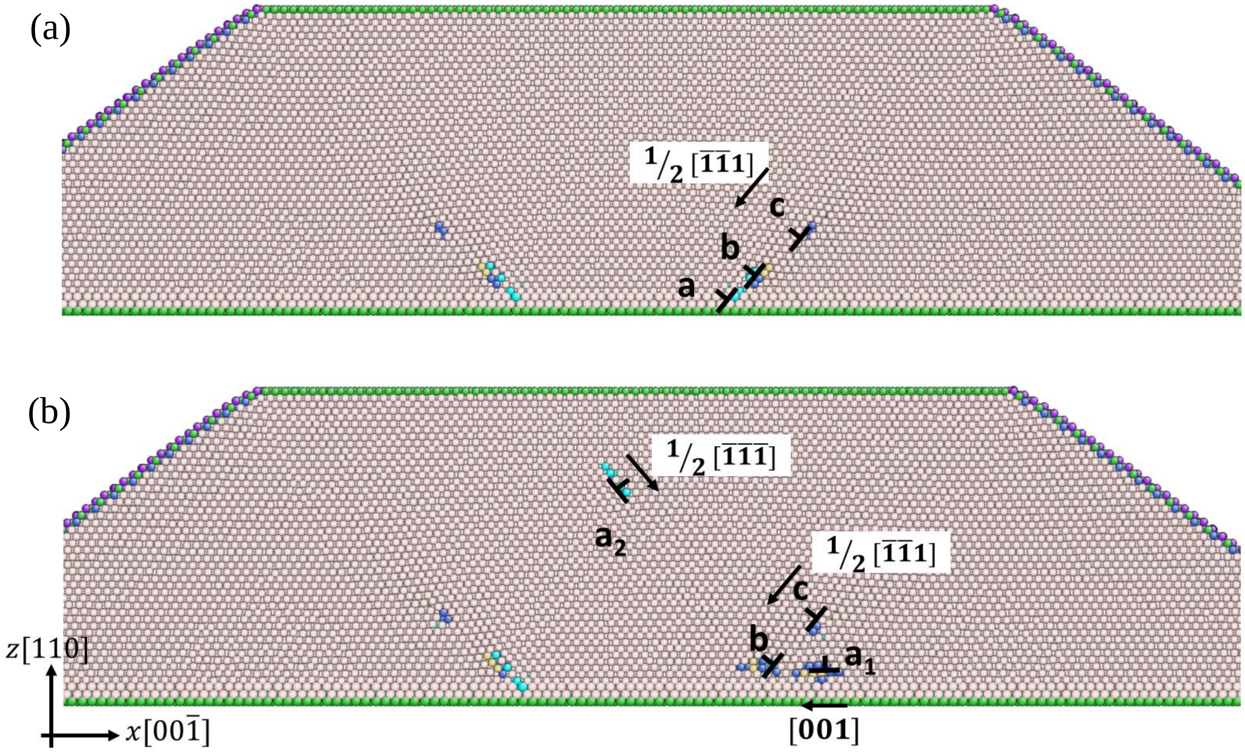

Fig. S6. The cross-split mechanisms in the nanowire. (**a**) The dislocation at the head of the pile-up splits (**b**) into ½$[\bar{1}\bar{1}\bar{1}]$ and [001] dislocations.

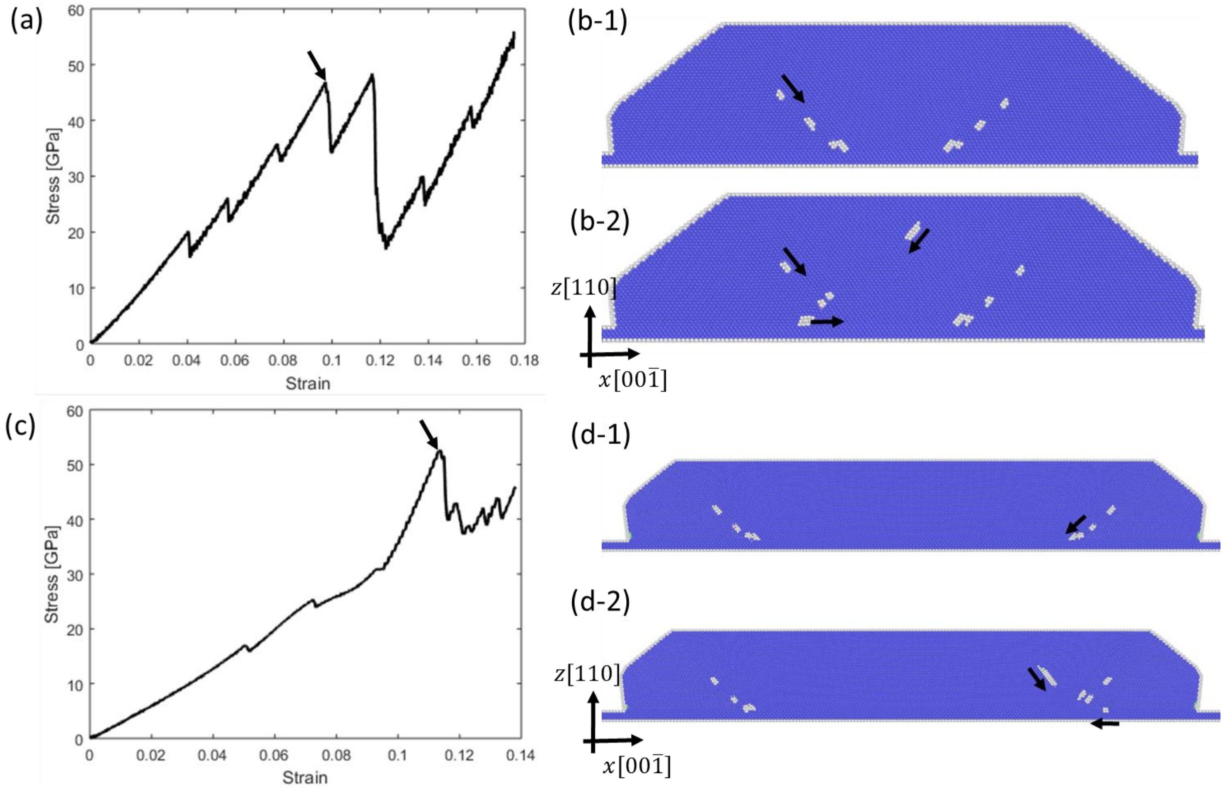

Fig. S7. The cross-split mechanisms captured with different interatomic potentials. (a) The stress-strain curve for a compression of a 9.5 high Fe nanowire, employing the Ackland *et al.*[5] potential. The dislocations inside the wire before and after cross-split, respectively, are shown in (b-1) and (b-2). To demonstrate the process for other BCC metals, Ta nanowire were compressed. (c) The stress-strain curve for compression of a wide 9.5 nm high Ta nanowire, employing EAM potential for Ta by Li et al[6]. (d-1) and (d-2) show the dislocations before and after cross-split.

<u>References</u>

1. Zysset, P. K., Edward Guo, X., Edward Hoffler, C., Moore, K. E. & Goldstein, S. A. Elastic modulus and hardness of cortical and trabecular bone lamellae measured by nanoindentation in the human femur. *J. Biomech.* **32,** 1005–1012 (1999).

2. Gregory V. Samsonov. Handbook of the Physicochemical Properties of the. *Springer US* 942 (1968). doi:0.1007/978-1-4684-6066-7

3. Hirth, J. & Lothe, J. Theory of Dislocations. (1982).

4. Terentyev, D. A., Osetsky, Y. N. & Bacon, D. J. Effects of temperature on structure and mobility of the〈 100〉 edge dislocation in body-centred cubic iron. *Acta Mater.* **58,** 2477–2482 (2010).

5. Ackland, G. J., Bacon, D. J., Calder, A. F. & Harry, T. Computer simulation of point defect properties in dilute Fe—Cu alloy using a many-body interatomic potential. *Philos. Mag. A* **75,** 713–732 (1997).

6. Li, Y., Siegel, D., Adams, J. & Liu, X.-Y. Embedded-atom-method tantalum potential developed by the force-matching method. *Phys. Rev. B* **67,** 1–8 (2003).
